# Supplementary material for: GWAS Enhances Genomic Prediction Accuracy of Caviar Yield, Caviar Color and Body Weight Traits in Sturgeons Using Whole-Genome Sequencing Data
Source: Int J Mol Sci. 2024 Sep 9;25(17):9756. doi: 10.3390/ijms25179756 (PMC11395957; doi:10.3390/ijms25179756)
Supplement: Supplementary file 1 [file ijms-25-09756-s001.zip › ijms-3165624-supplementary.pdf]

Table S1. Summary of statistics for whole-genome sequencing of 673 Russian sturgeons.

ID	Length(bp)	Raw reads	Raw base (G)	Sequencing depth (X)	Mapping rate
1	150;150	196,996,556	59.10	19.87	0.944253
2	150;150	192,110,615	57.63	19.8	0.943642
3	150;150	190,593,705	57.18	19.71	0.941928
4	150;150	193,073,751	57.92	19.48	0.943998
5	150;150	189,380,982	56.81	19.32	0.943517
6	150;150	189,171,028	56.75	19.29	0.943194
7	150;150	188,467,384	56.54	19.27	0.945271
8	150;150	189,210,756	56.76	19.23	0.943964
9	150;150	183,389,378	55.02	19.15	0.943117
10	150;150	184,491,783	55.35	18.67	0.9437
11	150;150	178,047,700	53.41	18.57	0.941217
12	150;150	185,619,561	55.69	18.47	0.945693
13	150;150	182,218,062	54.67	18.46	0.9429
14	150;150	180,142,564	54.04	18.43	0.942564
15	150;150	181,850,298	54.56	18.34	0.942171
16	150;150	179,356,565	53.81	18.32	0.942184
17	150;150	177,165,621	53.15	18.24	0.942074
18	150;150	180,874,818	54.26	18.18	0.941322
19	150;150	170,770,901	51.23	18.15	0.942788
20	150;150	173,403,354	52.02	18.02	0.943056
21	150;150	173,574,673	52.07	18	0.942629
22	150;150	166,606,653	49.98	17.96	0.94056
23	150;150	168,544,478	50.56	17.77	0.94212
24	150;150	167,727,712	50.32	17.75	0.939644
25	150;150	168,892,880	50.67	17.73	0.940612
26	150;150	170,296,535	51.09	17.73	0.94163
27	150;150	174,338,824	52.30	17.73	0.939217
28	150;150	171,583,877	51.48	17.65	0.941261
29	150;150	168,309,884	50.49	17.64	0.942995
30	150;150	170,546,563	51.16	17.64	0.942568
31	150;150	168,218,470	50.47	17.6	0.941841
32	150;150	165,950,234	49.79	17.51	0.946256
33	150;150	168,108,532	50.43	17.49	0.941747
34	150;150	165,973,233	49.79	17.47	0.941901
35	150;150	168,162,399	50.45	17.45	0.939733
36	150;150	172,447,463	51.73	17.42	0.942198
37	150;150	167,640,968	50.29	17.28	0.940054
38	150;150	168,660,384	50.60	17.16	0.939735
39	150;150	168,689,909	50.61	17.16	0.939863
40	150;150	167,984,804	50.40	17.1	0.938483
41	150;150	168,494,459	50.55	17.09	0.940941
42	150;150	166,732,662	50.02	17.04	0.940864

43	150;150	168,048,028	50.41	16.96	0.938846
44	150;150	175,465,414	52.64	14.82	0.934694
45	150;150	102,564,949	30.77	14.9	0.931999
46	150;150	74,828,519	22.45	10.89	0.920085
47	150;150	183,431,437	55.03	25.85	0.939178
48	150;150	175,321,867	52.60	24.82	0.938729
49	150;150	172,961,978	51.89	24.34	0.937333
50	150;150	169,057,219	50.72	23.76	0.936497
51	150;150	160,331,181	48.10	23.38	0.937973
52	150;150	161,900,093	48.57	23.27	0.936886
53	150;150	164,857,392	49.46	23.26	0.943357
54	150;150	155,792,992	46.74	22.16	0.935232
55	150;150	141,839,425	42.55	20.97	0.936025
56	150;150	145,140,638	43.54	20.7	0.939069
57	150;150	136,937,786	41.08	19.43	0.931319
58	150;150	128,489,626	38.55	19.09	0.934448
59	150;150	132,623,967	39.79	18.97	0.928688
60	150;150	131,292,112	39.39	18.7	0.934784
61	150;150	127,315,771	38.19	18.61	0.933933
62	150;150	126,756,776	38.03	18.45	0.932004
63	150;150	125,378,840	37.61	18.29	0.9315
64	150;150	129,154,277	38.75	18.2	0.939446
65	150;150	124,217,377	37.27	18.11	0.935652
66	150;150	124,089,604	37.23	17.89	0.9349
67	150;150	121,523,801	36.46	17.88	0.934842
68	150;150	121,416,164	36.42	17.8	0.932433
69	150;150	119,837,015	35.95	17.8	0.933634
70	150;150	121,798,648	36.54	17.72	0.933775
71	150;150	121,544,025	36.46	17.71	0.933675
72	150;150	121,290,367	36.39	17.7	0.937557
73	150;150	121,866,920	36.56	17.7	0.936367
74	150;150	120,866,743	36.26	17.68	0.93512
75	150;150	120,207,804	36.06	17.63	0.934287
76	150;150	120,011,633	36.00	17.61	0.93434
77	150;150	119,908,810	35.97	17.46	0.934991
78	150;150	118,833,530	35.65	17.46	0.935128
79	150;150	118,164,072	35.45	17.43	0.933439
80	150;150	119,525,389	35.86	17.41	0.935566
81	150;150	118,697,248	35.61	17.33	0.929135
82	150;150	116,415,561	34.92	17.29	0.932636
83	150;150	118,512,916	35.55	17.13	0.934453
84	150;150	122,488,382	36.75	17.12	0.92795
85	150;150	118,369,420	35.51	16.94	0.927335
86	150;150	117,008,466	35.10	16.88	0.933883

87	150;150	114,549,535	34.36	16.85	0.932296
88	150;150	114,981,686	34.49	16.8	0.932423
89	150;150	113,103,485	33.93	16.77	0.932355
90	150;150	117,255,514	35.18	16.74	0.935663
91	150;150	113,444,326	34.03	16.68	0.933596
92	150;150	114,447,566	34.33	16.67	0.933604
93	150;150	115,360,593	34.61	16.53	0.929794
94	150;150	111,069,047	33.32	16.49	0.931987
95	150;150	111,797,159	33.54	16.42	0.933373
96	150;150	111,611,885	33.48	16.4	0.931157
97	150;150	112,280,756	33.68	16.37	0.931586
98	150;150	111,951,421	33.59	16.36	0.933222
99	150;150	114,785,817	34.44	16.34	0.931426
100	150;150	111,069,947	33.32	16.34	0.930912
101	150;150	111,152,018	33.35	16.31	0.933738
102	150;150	115,707,904	34.71	16.3	0.934475
103	150;150	113,229,520	33.97	16.25	0.936704
104	150;150	111,627,455	33.49	16.23	0.933602
105	150;150	111,572,761	33.47	16.22	0.932346
106	150;150	111,809,819	33.54	16.22	0.934662
107	150;150	109,561,289	32.87	16.2	0.929776
108	150;150	110,491,841	33.15	16.2	0.933888
109	150;150	112,543,714	33.76	16.2	0.937341
110	150;150	110,065,744	33.02	16.17	0.932722
111	150;150	110,377,482	33.11	16.17	0.931432
112	150;150	112,635,807	33.79	16.15	0.935859
113	150;150	110,142,046	33.04	16.11	0.934607
114	150;150	110,184,805	33.06	16.11	0.931147
115	150;150	107,887,725	32.37	16.1	0.929938
116	150;150	109,810,530	32.94	16.08	0.931803
117	150;150	108,294,315	32.49	16.07	0.933242
118	150;150	110,104,021	33.03	16.06	0.933144
119	150;150	109,353,241	32.81	16.06	0.932167
120	150;150	111,176,395	33.35	16.02	0.934059
121	150;150	109,711,734	32.91	16.02	0.932593
122	150;150	110,310,969	33.09	16	0.93483
123	150;150	108,941,003	32.68	15.98	0.932354
124	150;150	107,684,697	32.31	15.92	0.931936
125	150;150	108,994,846	32.70	15.91	0.932841
126	150;150	110,514,153	33.15	15.82	0.935682
127	150;150	107,720,284	32.32	15.82	0.933562
128	150;150	107,360,834	32.21	15.77	0.931904
129	150;150	107,715,052	32.31	15.77	0.932369
130	150;150	107,852,844	32.36	15.75	0.932221

131	150;150	106,366,938	31.91	15.74	0.932395
132	150;150	108,365,156	32.51	15.74	0.930864
133	150;150	107,787,225	32.34	15.74	0.933684
134	150;150	107,227,902	32.17	15.72	0.93047
135	150;150	107,119,055	32.14	15.71	0.931697
136	150;150	107,186,152	32.16	15.69	0.9321
137	150;150	109,750,893	32.93	15.66	0.934465
138	150;150	105,962,007	31.79	15.61	0.93013
139	150;150	105,909,108	31.77	15.6	0.931652
140	150;150	106,829,860	32.05	15.58	0.931659
141	150;150	105,679,888	31.70	15.54	0.93101
142	150;150	105,898,828	31.77	15.52	0.928395
143	150;150	104,526,612	31.36	15.51	0.930935
144	150;150	104,335,994	31.30	15.51	0.932615
145	150;150	105,441,060	31.63	15.48	0.93209
146	150;150	107,002,630	32.10	15.46	0.932549
147	150;150	104,545,910	31.36	15.45	0.931618
148	150;150	104,898,941	31.47	15.44	0.932552
149	150;150	104,885,357	31.47	15.39	0.930609
150	150;150	105,684,454	31.71	15.39	0.931814
151	150;150	106,400,829	31.92	15.38	0.929987
152	150;150	104,979,201	31.49	15.37	0.932353
153	150;150	102,883,352	30.87	15.36	0.927588
154	150;150	105,377,834	31.61	15.36	0.932938
155	150;150	104,857,115	31.46	15.36	0.93117
156	150;150	105,155,177	31.55	15.34	0.932694
157	150;150	103,368,345	31.01	15.33	0.931785
158	150;150	104,564,188	31.37	15.33	0.93136
159	150;150	102,880,157	30.86	15.3	0.933603
160	150;150	104,628,531	31.39	15.29	0.930621
161	150;150	105,024,958	31.51	15.28	0.931147
162	150;150	103,828,707	31.15	15.26	0.932297
163	150;150	103,267,174	30.98	15.24	0.930728
164	150;150	103,454,189	31.04	15.23	0.932182
165	150;150	104,029,431	31.21	15.23	0.931256
166	150;150	104,925,619	31.48	15.21	0.926098
167	150;150	103,297,999	30.99	15.2	0.929621
168	150;150	103,134,191	30.94	15.19	0.931986
169	150;150	102,094,129	30.63	15.16	0.929893
170	150;150	102,847,766	30.85	15.16	0.929111
171	150;150	102,011,271	30.60	15.13	0.930396
172	150;150	104,658,073	31.40	15.12	0.934182
173	150;150	102,894,868	30.87	15.1	0.930207
174	150;150	102,991,934	30.90	15.09	0.931598

175	150;150	102,788,177	30.84	15.06	0.930093
176	150;150	104,847,456	31.45	15.05	0.927371
177	150;150	104,605,276	31.38	15.04	0.924854
178	150;150	102,800,634	30.84	15.03	0.932312
179	150;150	102,320,470	30.70	15.03	0.930495
180	150;150	102,913,296	30.87	15.03	0.930953
181	150;150	104,170,650	31.25	15.02	0.931666
182	150;150	106,604,445	31.98	15.01	0.934649
183	150;150	102,057,875	30.62	15.01	0.931254
184	150;150	102,523,330	30.76	15.01	0.930888
185	150;150	102,365,245	30.71	14.99	0.931299
186	150;150	102,009,747	30.60	14.94	0.928419
187	150;150	101,877,079	30.56	14.94	0.931023
188	150;150	100,745,617	30.22	14.94	0.931926
189	150;150	105,155,403	31.55	14.94	0.931703
190	150;150	101,387,535	30.42	14.93	0.931048
191	150;150	103,841,285	31.15	14.92	0.933601
192	150;150	102,854,108	30.86	14.9	0.92502
193	150;150	100,113,997	30.03	14.9	0.929725
194	150;150	100,450,200	30.14	14.9	0.928967
195	150;150	104,730,137	31.42	14.89	0.933594
196	150;150	105,949,677	31.78	14.88	0.933238
197	150;150	101,062,536	30.32	14.87	0.930448
198	150;150	100,689,060	30.21	14.86	0.931204
199	150;150	101,695,407	30.51	14.84	0.922959
200	150;150	102,266,031	30.68	14.83	0.931835
201	150;150	100,984,664	30.30	14.83	0.929883
202	150;150	102,929,000	30.88	14.83	0.932991
203	150;150	102,892,990	30.87	14.8	0.932578
204	150;150	102,026,701	30.61	14.8	0.925288
205	150;150	99,362,636	29.81	14.8	0.926641
206	150;150	99,870,140	29.96	14.79	0.928577
207	150;150	99,251,349	29.78	14.79	0.928918
208	150;150	99,622,596	29.89	14.78	0.930338
209	150;150	101,159,769	30.35	14.77	0.930223
210	150;150	100,807,185	30.24	14.76	0.93017
211	150;150	101,305,869	30.39	14.76	0.930903
212	150;150	99,147,594	29.74	14.74	0.931216
213	150;150	101,339,387	30.40	14.74	0.928319
214	150;150	100,912,547	30.27	14.72	0.93284
215	150;150	98,966,925	29.69	14.71	0.930212
216	150;150	101,749,334	30.52	14.68	0.932389
217	150;150	100,808,246	30.24	14.68	0.930478
218	150;150	99,809,403	29.94	14.66	0.930485

219	150;150	100,027,532	30.01	14.66	0.936639
220	150;150	103,091,051	30.93	14.65	0.930687
221	150;150	101,802,391	30.54	14.65	0.928229
222	150;150	101,799,307	30.54	14.62	0.933211
223	150;150	100,578,827	30.17	14.62	0.924339
224	150;150	100,677,713	30.20	14.61	0.929958
225	150;150	99,155,994	29.75	14.61	0.928599
226	150;150	98,367,450	29.51	14.61	0.928845
227	150;150	101,730,635	30.52	14.6	0.931071
228	150;150	98,720,812	29.62	14.6	0.929104
229	150;150	99,121,932	29.74	14.59	0.930854
230	150;150	99,205,297	29.76	14.58	0.928606
231	150;150	98,220,923	29.47	14.58	0.929528
232	150;150	101,674,257	30.50	14.58	0.935958
233	150;150	99,946,299	29.98	14.58	0.930438
234	150;150	98,887,387	29.67	14.57	0.928497
235	150;150	100,055,772	30.02	14.56	0.929716
236	150;150	100,116,941	30.04	14.56	0.929835
237	150;150	99,620,693	29.89	14.56	0.930837
238	150;150	98,998,373	29.70	14.55	0.930143
239	150;150	101,460,875	30.44	14.55	0.932712
240	150;150	99,057,684	29.72	14.54	0.929587
241	150;150	101,027,708	30.31	14.54	0.931654
242	150;150	99,724,666	29.92	14.53	0.930811
243	150;150	98,873,862	29.66	14.52	0.9325
244	150;150	98,532,703	29.56	14.52	0.930101
245	150;150	99,545,108	29.86	14.52	0.931406
246	150;150	98,649,796	29.59	14.51	0.930727
247	150;150	99,481,273	29.84	14.49	0.923296
248	150;150	99,447,381	29.83	14.49	0.922643
249	150;150	100,606,966	30.18	14.48	0.933177
250	150;150	98,280,271	29.48	14.48	0.929305
251	150;150	98,562,799	29.57	14.47	0.930487
252	150;150	98,567,531	29.57	14.47	0.929312
253	150;150	97,561,532	29.27	14.46	0.930123
254	150;150	100,002,410	30.00	14.46	0.9287
255	150;150	97,023,330	29.11	14.44	0.926877
256	150;150	98,926,176	29.68	14.41	0.930239
257	150;150	100,558,811	30.17	14.4	0.930954
258	150;150	99,897,219	29.97	14.4	0.931711
259	150;150	98,910,004	29.67	14.4	0.931096
260	150;150	99,700,972	29.91	14.39	0.928941
261	150;150	98,813,288	29.64	14.38	0.927771
262	150;150	98,655,790	29.60	14.37	0.923788

263	150;150	98,300,136	29.49	14.35	0.92922
264	150;150	98,253,041	29.48	14.34	0.930737
265	150;150	97,497,078	29.25	14.33	0.928841
266	150;150	97,676,993	29.30	14.32	0.930095
267	150;150	97,731,966	29.32	14.32	0.929054
268	150;150	96,669,841	29.00	14.31	0.92942
269	150;150	100,041,454	30.01	14.29	0.931571
270	150;150	98,718,105	29.62	14.29	0.930231
271	150;150	97,545,298	29.26	14.27	0.929999
272	150;150	96,779,117	29.03	14.27	0.926338
273	150;150	99,214,726	29.76	14.27	0.9335
274	150;150	96,922,475	29.08	14.23	0.928293
275	150;150	97,244,156	29.17	14.23	0.927152
276	150;150	96,254,517	28.88	14.23	0.928569
277	150;150	96,208,658	28.86	14.22	0.929585
278	150;150	95,164,887	28.55	14.22	0.92612
279	150;150	96,334,726	28.90	14.21	0.928516
280	150;150	96,904,127	29.07	14.21	0.928976
281	150;150	101,171,439	30.35	14.2	0.933341
282	150;150	96,560,830	28.97	14.2	0.93003
283	150;150	95,407,970	28.62	14.19	0.928489
284	150;150	97,311,093	29.19	14.19	0.923382
285	150;150	96,255,013	28.88	14.18	0.929809
286	150;150	95,692,015	28.71	14.18	0.928875
287	150;150	97,210,413	29.16	14.18	0.931136
288	150;150	97,744,800	29.32	14.17	0.92856
289	150;150	95,701,578	28.71	14.17	0.925291
290	150;150	98,305,846	29.49	14.16	0.935049
291	150;150	96,077,123	28.82	14.15	0.928391
292	150;150	98,235,768	29.47	14.13	0.927711
293	150;150	95,179,006	28.55	14.12	0.928773
294	150;150	96,474,685	28.94	14.11	0.929125
295	150;150	96,566,841	28.97	14.11	0.929301
296	150;150	95,688,926	28.71	14.1	0.929488
297	150;150	96,554,649	28.97	14.1	0.929753
298	150;150	95,511,539	28.65	14.1	0.928752
299	150;150	96,080,104	28.82	14.09	0.927159
300	150;150	96,714,619	29.01	14.09	0.923361
301	150;150	97,254,864	29.18	14.09	0.931469
302	150;150	95,757,204	28.73	14.08	0.927453
303	150;150	95,505,423	28.65	14.08	0.929746
304	150;150	95,501,144	28.65	14.06	0.930055
305	150;150	94,358,265	28.31	14.06	0.928405
306	150;150	95,741,054	28.72	14.05	0.929562

307	150;150	95,248,898	28.57	14.04	0.929062
308	150;150	97,638,604	29.29	14.04	0.928855
309	150;150	95,577,252	28.67	14.03	0.927693
310	150;150	98,208,909	29.46	14.03	0.93341
311	150;150	94,702,521	28.41	14.02	0.928852
312	150;150	94,797,627	28.44	14.02	0.926651
313	150;150	95,200,671	28.56	14.02	0.927708
314	150;150	94,554,232	28.37	14.01	0.9267
315	150;150	96,340,420	28.90	14.01	0.933354
316	150;150	95,763,979	28.73	14.01	0.928789
317	150;150	96,081,849	28.82	14	0.928858
318	150;150	93,989,411	28.20	13.98	0.929143
319	150;150	94,708,723	28.41	13.97	0.929087
320	150;150	95,198,717	28.56	13.96	0.929664
321	150;150	94,416,552	28.32	13.95	0.928817
322	150;150	94,916,878	28.48	13.95	0.92742
323	150;150	93,725,748	28.12	13.94	0.928774
324	150;150	95,038,578	28.51	13.93	0.927689
325	150;150	94,752,655	28.43	13.91	0.928799
326	150;150	95,680,545	28.70	13.9	0.935864
327	150;150	96,668,575	29.00	13.88	0.931492
328	150;150	96,912,010	29.07	13.86	0.931688
329	150;150	94,144,099	28.24	13.86	0.927997
330	150;150	95,043,432	28.51	13.86	0.922436
331	150;150	96,786,082	29.04	13.85	0.931112
332	150;150	93,196,342	27.96	13.83	0.928205
333	150;150	95,672,901	28.70	13.82	0.931281
334	150;150	93,236,954	27.97	13.82	0.928298
335	150;150	94,694,809	28.41	13.82	0.931568
336	150;150	95,289,339	28.59	13.81	0.931054
337	150;150	94,590,466	28.38	13.79	0.921608
338	150;150	93,700,848	28.11	13.78	0.922785
339	150;150	95,910,627	28.77	13.78	0.930396
340	150;150	95,907,958	28.77	13.78	0.930396
341	150;150	92,891,043	27.87	13.78	0.930026
342	150;150	92,674,117	27.80	13.75	0.925761
343	150;150	93,715,009	28.11	13.72	0.928805
344	150;150	94,046,411	28.21	13.72	0.929753
345	150;150	95,116,102	28.53	13.71	0.930175
346	150;150	94,476,639	28.34	13.7	0.93099
347	150;150	95,053,931	28.52	13.7	0.929813
348	150;150	93,026,530	27.91	13.7	0.929006
349	150;150	91,901,910	27.57	13.69	0.928195
350	150;150	92,986,400	27.90	13.68	0.928246

351	150;150	94,013,419	28.20	13.68	0.929121
352	150;150	94,945,738	28.48	13.67	0.932065
353	150;150	93,956,105	28.19	13.67	0.927905
354	150;150	93,364,607	28.01	13.67	0.928504
355	150;150	93,256,111	27.98	13.67	0.928053
356	150;150	93,331,319	28.00	13.67	0.928425
357	150;150	93,504,334	28.05	13.65	0.927648
358	150;150	93,616,413	28.08	13.64	0.921615
359	150;150	92,237,773	27.67	13.64	0.927002
360	150;150	91,869,484	27.56	13.63	0.930097
361	150;150	93,260,124	27.98	13.62	0.919975
362	150;150	91,840,489	27.55	13.62	0.928496
363	150;150	92,709,921	27.81	13.62	0.928332
364	150;150	91,360,182	27.41	13.62	0.934157
365	150;150	92,797,251	27.84	13.62	0.926354
366	150;150	93,090,387	27.93	13.59	0.92804
367	150;150	93,210,439	27.96	13.59	0.929666
368	150;150	92,710,399	27.81	13.57	0.92769
369	150;150	94,094,648	28.23	13.57	0.930652
370	150;150	92,871,868	27.86	13.56	0.92803
371	150;150	92,332,389	27.70	13.55	0.929783
372	150;150	91,823,912	27.55	13.54	0.927329
373	150;150	93,421,843	28.03	13.54	0.927551
374	150;150	93,183,782	27.96	13.53	0.928351
375	150;150	91,920,207	27.58	13.51	0.928598
376	150;150	90,793,040	27.24	13.5	0.928668
377	150;150	92,222,731	27.67	13.5	0.928571
378	150;150	91,503,960	27.45	13.49	0.927653
379	150;150	91,110,202	27.33	13.48	0.928577
380	150;150	93,906,684	28.17	13.48	0.929314
381	150;150	91,177,279	27.35	13.47	0.927762
382	150;150	91,605,219	27.48	13.47	0.926618
383	150;150	90,759,170	27.23	13.47	0.928816
384	150;150	90,963,427	27.29	13.44	0.925387
385	150;150	91,555,010	27.47	13.44	0.927352
386	150;150	91,599,915	27.48	13.43	0.926363
387	150;150	91,839,063	27.55	13.43	0.928043
388	150;150	90,544,087	27.16	13.43	0.927898
389	150;150	91,373,798	27.41	13.42	0.927171
390	150;150	90,927,380	27.28	13.42	0.927003
391	150;150	91,065,358	27.32	13.42	0.928386
392	150;150	91,798,710	27.54	13.41	0.926456
393	150;150	92,011,559	27.60	13.41	0.927454
394	150;150	92,724,670	27.82	13.4	0.928502

395	150;150	91,578,285	27.47	13.39	0.92783
396	150;150	93,940,334	28.18	13.39	0.930386
397	150;150	91,306,768	27.39	13.37	0.927053
398	150;150	90,423,838	27.13	13.35	0.926709
399	150;150	93,122,611	27.94	13.32	0.929839
400	150;150	91,076,804	27.32	13.31	0.927332
401	150;150	90,834,871	27.25	13.3	0.925289
402	150;150	90,064,669	27.02	13.26	0.927583
403	150;150	91,621,526	27.49	13.26	0.927747
404	150;150	91,598,813	27.48	13.26	0.928321
405	150;150	90,455,098	27.14	13.22	0.925525
406	150;150	90,095,647	27.03	13.22	0.926364
407	150;150	89,293,276	26.79	13.2	0.927892
408	150;150	89,271,449	26.78	13.2	0.929463
409	150;150	90,737,952	27.22	13.19	0.920908
410	150;150	89,311,232	26.79	13.18	0.927641
411	150;150	91,337,559	27.40	13.18	0.92938
412	150;150	90,954,939	27.29	13.18	0.923664
413	150;150	89,497,033	26.85	13.14	0.926898
414	150;150	91,369,180	27.41	13.14	0.929566
415	150;150	89,787,374	26.94	13.12	0.926765
416	150;150	91,485,218	27.45	13.11	0.92898
417	150;150	89,979,278	26.99	13.11	0.92484
418	150;150	89,470,008	26.84	13.1	0.925517
419	150;150	87,832,021	26.35	13.1	0.926077
420	150;150	89,359,350	26.81	13.09	0.923352
421	150;150	88,920,811	26.68	13.09	0.925827
422	150;150	89,804,111	26.94	13.07	0.926353
423	150;150	89,866,546	26.96	13.07	0.924888
424	150;150	88,469,235	26.54	13.06	0.925493
425	150;150	89,796,396	26.94	13.05	0.925077
426	150;150	90,479,382	27.14	13.05	0.929707
427	150;150	89,171,367	26.75	13.05	0.926935
428	150;150	90,534,809	27.16	13.03	0.929597
429	150;150	88,745,985	26.62	13.03	0.925506
430	150;150	88,789,455	26.64	13.02	0.926738
431	150;150	88,959,661	26.69	13.01	0.927816
432	150;150	89,569,885	26.87	13	0.927474
433	150;150	93,497,501	28.05	12.93	0.931117
434	150;150	88,412,275	26.52	12.93	0.926858
435	150;150	87,852,342	26.36	12.91	0.924136
436	150;150	87,181,481	26.15	12.91	0.924423
437	150;150	87,748,743	26.32	12.89	0.924544
438	150;150	88,177,433	26.45	12.89	0.925379

439	150;150	90,128,091	27.04	12.88	0.925415
440	150;150	87,259,383	26.18	12.87	0.926408
441	150;150	87,006,988	26.10	12.87	0.924568
442	150;150	87,837,963	26.35	12.87	0.927397
443	150;150	86,479,314	25.94	12.85	0.924373
444	150;150	87,324,374	26.20	12.82	0.926031
445	150;150	86,494,423	25.95	12.81	0.927348
446	150;150	88,613,034	26.58	12.81	0.928213
447	150;150	86,712,850	26.01	12.78	0.924127
448	150;150	87,568,589	26.27	12.78	0.927328
449	150;150	85,962,220	25.79	12.77	0.926179
450	150;150	86,395,421	25.92	12.77	0.925792
451	150;150	87,164,770	26.15	12.77	0.926391
452	150;150	86,824,786	26.05	12.76	0.926325
453	150;150	88,200,390	26.46	12.75	0.929232
454	150;150	87,273,725	26.18	12.75	0.926042
455	150;150	85,644,626	25.69	12.71	0.926539
456	150;150	87,518,037	26.26	12.69	0.917075
457	150;150	86,500,898	25.95	12.67	0.925204
458	150;150	84,918,076	25.48	12.65	0.924407
459	150;150	85,109,121	25.53	12.63	0.927237
460	150;150	84,773,933	25.43	12.63	0.926586
461	150;150	84,795,797	25.44	12.58	0.926248
462	150;150	85,672,758	25.70	12.58	0.923729
463	150;150	85,203,711	25.56	12.54	0.926156
464	150;150	84,242,700	25.27	12.54	0.923639
465	150;150	84,436,996	25.33	12.54	0.926876
466	150;150	84,099,444	25.23	12.47	0.926175
467	150;150	86,560,344	25.97	12.39	0.926199
468	150;150	85,321,284	25.60	12.37	0.925187
469	150;150	85,294,843	25.59	12.37	0.926835
470	150;150	83,920,271	25.18	12.36	0.925306
471	150;150	84,225,807	25.27	12.35	0.924523
472	150;150	83,661,314	25.10	12.35	0.925464
473	150;150	83,293,770	24.99	12.34	0.925502
474	150;150	83,161,915	24.95	12.34	0.924229
475	150;150	84,584,416	25.38	12.33	0.918311
476	150;150	83,681,377	25.10	12.29	0.924881
477	150;150	84,791,552	25.44	12.28	0.927612
478	150;150	83,554,024	25.07	12.28	0.925036
479	150;150	83,691,845	25.11	12.27	0.923795
480	150;150	82,890,732	24.87	12.26	0.925352
481	150;150	83,261,873	24.98	12.25	0.925045
482	150;150	84,246,528	25.27	12.18	0.925063

483	150;150	82,140,365	24.64	12.18	0.925066
484	150;150	84,394,095	25.32	12.17	0.92573
485	150;150	82,406,787	24.72	12.17	0.923167
486	150;150	82,716,082	24.81	12.14	0.924574
487	150;150	82,294,230	24.69	12.1	0.924354
488	150;150	81,411,141	24.42	12.05	0.923002
489	150;150	81,917,289	24.58	12.04	0.92419
490	150;150	81,847,936	24.55	11.97	0.923871
491	150;150	80,813,115	24.24	11.96	0.9257
492	150;150	81,067,659	24.32	11.92	0.921339
493	150;150	84,027,277	25.21	11.89	0.926148
494	150;150	80,753,890	24.23	11.88	0.918938
495	150;150	80,215,564	24.06	11.88	0.923108
496	150;150	81,001,304	24.30	11.84	0.916403
497	150;150	80,526,183	24.16	11.84	0.919896
498	150;150	80,450,173	24.14	11.82	0.92679
499	150;150	79,467,315	23.84	11.79	0.925384
500	150;150	80,984,381	24.30	11.78	0.916767
501	150;150	83,393,739	25.02	11.77	0.92771
502	150;150	79,695,174	23.91	11.76	0.924662
503	150;150	79,741,161	23.92	11.73	0.915573
504	150;150	81,125,781	24.34	11.73	0.92335
505	150;150	80,135,215	24.04	11.73	0.923649
506	150;150	79,579,997	23.87	11.71	0.923294
507	150;150	78,453,179	23.54	11.69	0.922895
508	150;150	80,076,114	24.02	11.69	0.92339
509	150;150	79,067,738	23.72	11.65	0.919478
510	150;150	80,952,188	24.29	11.62	0.925288
511	150;150	79,697,105	23.91	11.61	0.925858
512	150;150	78,453,316	23.54	11.58	0.922462
513	150;150	78,395,155	23.52	11.55	0.927488
514	150;150	78,744,176	23.62	11.53	0.922423
515	150;150	78,436,824	23.53	11.52	0.922811
516	150;150	79,256,037	23.78	11.52	0.91653
517	150;150	78,560,784	23.57	11.51	0.922293
518	150;150	79,479,933	23.84	11.5	0.922045
519	150;150	78,440,556	23.53	11.46	0.924606
520	150;150	78,464,746	23.54	11.45	0.922982
521	150;150	77,957,093	23.39	11.43	0.918768
522	150;150	77,683,917	23.31	11.41	0.922861
523	150;150	77,710,612	23.31	11.36	0.923111
524	150;150	77,362,749	23.21	11.36	0.92036
525	150;150	77,400,357	23.22	11.35	0.919787
526	150;150	76,860,401	23.06	11.34	0.917901

527	150;150	77,514,377	23.25	11.34	0.922452
528	150;150	77,835,415	23.35	11.33	0.923814
529	150;150	77,016,336	23.10	11.31	0.919645
530	150;150	77,091,192	23.13	11.3	0.922101
531	150;150	75,846,530	22.75	11.28	0.919472
532	150;150	76,620,544	22.99	11.28	0.91972
533	150;150	76,495,862	22.95	11.27	0.918864
534	150;150	76,379,755	22.91	11.26	0.923056
535	150;150	77,811,500	23.34	11.25	0.921169
536	150;150	77,119,415	23.14	11.24	0.92836
537	150;150	76,281,040	22.88	11.23	0.922738
538	150;150	76,171,661	22.85	11.21	0.918804
539	150;150	76,488,946	22.95	11.21	0.917205
540	150;150	74,790,366	22.44	11.15	0.92052
541	150;150	76,680,407	23.00	11.1	0.914501
542	150;150	75,115,026	22.53	11.05	0.917096
543	150;150	75,042,824	22.51	11.04	0.921307
544	150;150	74,269,773	22.28	11.03	0.92196
545	150;150	74,273,132	22.28	11.03	0.921204
546	150;150	77,946,494	23.38	11.02	0.920753
547	150;150	74,804,312	22.44	11	0.921214
548	150;150	74,720,863	22.42	10.98	0.917238
549	150;150	74,709,448	22.41	10.95	0.916778
550	150;150	73,966,844	22.19	10.93	0.91676
551	150;150	73,381,758	22.01	10.91	0.921459
552	150;150	74,051,543	22.22	10.88	0.916744
553	150;150	73,995,562	22.20	10.86	0.917166
554	150;150	73,763,718	22.13	10.84	0.916348
555	150;150	75,595,959	22.68	10.83	0.921553
556	150;150	73,519,661	22.06	10.8	0.916771
557	150;150	75,770,081	22.73	10.77	0.921395
558	150;150	73,522,942	22.06	10.76	0.915498
559	150;150	74,359,344	22.31	10.74	0.922104
560	150;150	72,844,591	21.85	10.73	0.919711
561	150;150	72,013,657	21.60	10.64	0.915576
562	150;150	73,427,845	22.03	10.6	0.918685
563	150;150	73,255,964	21.98	10.59	0.922019
564	150;150	71,559,890	21.47	10.56	0.920555
565	150;150	72,234,997	21.67	10.56	0.916167
566	150;150	72,021,753	21.61	10.56	0.91989
567	150;150	71,527,869	21.46	10.54	0.914751
568	150;150	73,639,628	22.09	10.54	0.923182
569	150;150	72,140,858	21.64	10.5	0.916258
570	150;150	71,447,602	21.43	10.5	0.919883

571	150;150	71,843,335	21.55	10.49	0.918855
572	150;150	71,003,634	21.30	10.44	0.921878
573	150;150	72,468,875	21.74	10.43	0.918608
574	150;150	73,269,302	21.98	10.42	0.919951
575	150;150	70,678,273	21.20	10.38	0.920091
576	150;150	71,188,702	21.36	10.37	0.915783
577	150;150	71,161,854	21.35	10.3	0.915571
578	150;150	69,880,063	20.96	10.3	0.916061
579	150;150	72,212,176	21.66	10.3	0.923716
580	150;150	70,825,414	21.25	10.29	0.917444
581	150;150	69,737,758	20.92	10.29	0.916981
582	150;150	69,013,651	20.70	10.21	0.918603
583	150;150	71,115,234	21.33	10.19	0.923855
584	150;150	69,233,911	20.77	10.18	0.919217
585	150;150	69,309,790	20.79	10.11	0.919237
586	150;150	68,571,306	20.57	10.04	0.918065
587	150;150	68,410,135	20.52	10.04	0.92137
588	150;150	69,464,860	20.84	10.04	0.918201
589	150;150	68,156,731	20.45	10.03	0.913119
590	150;150	68,240,486	20.47	10.02	0.914521
591	150;150	67,394,547	20.22	9.94	0.913042
592	150;150	67,123,088	20.14	9.88	0.917875
593	150;150	190,699,686	28.60	13.41	0.928118
594	150;150	114,049,764	17.11	8.05	0.90224
595	150;150	111,765,900	16.76	7.78	0.900881
596	150;150	109,579,312	16.44	7.65	0.902894
597	150;150	109,316,758	16.40	7.62	0.901943
598	150;150	108,141,570	16.22	7.56	0.90258
599	150;150	105,991,900	15.90	7.44	0.905572
600	150;150	107,780,028	16.17	7.43	0.901908
601	150;150	104,662,420	15.70	7.32	0.898854
602	150;150	104,053,438	15.61	7.25	0.900528
603	150;150	100,716,644	15.11	7.01	0.896348
604	150;150	99,992,078	15.00	7	0.896608
605	150;150	99,642,170	14.95	6.94	0.897324
606	150;150	98,188,912	14.73	6.84	0.894429
607	150;150	96,807,120	14.52	6.73	0.900622
608	150;150	97,300,160	14.60	6.73	0.89252
609	150;150	95,310,002	14.30	6.62	0.891024
610	150;150	95,054,036	14.26	6.6	0.892663
611	150;150	94,112,618	14.12	6.56	0.893351
612	150;150	93,703,642	14.06	6.56	0.890428
613	150;150	94,316,956	14.15	6.54	0.892431
614	150;150	93,510,874	14.03	6.53	0.893394

615	150;150	93,517,160	14.03	6.51	0.894767
616	150;150	91,878,568	13.78	6.45	0.887832
617	150;150	90,695,106	13.60	6.36	0.895742
618	150;150	89,181,990	13.38	6.35	0.892444
619	150;150	90,356,128	13.55	6.33	0.894527
620	150;150	90,908,656	13.64	6.26	0.891553
621	150;150	89,642,048	13.45	6.23	0.885981
622	150;150	90,136,902	13.52	6.22	0.888079
623	150;150	88,028,656	13.20	6.21	0.886898
624	150;150	87,648,274	13.15	6.08	0.890042
625	150;150	86,572,584	12.99	6.06	0.886235
626	150;150	86,397,062	12.96	6	0.886487
627	150;150	85,007,716	12.75	5.96	0.889706
628	150;150	84,921,750	12.74	5.92	0.884462
629	150;150	83,668,176	12.55	5.81	0.88442
630	150;150	82,855,678	12.43	5.77	0.887771
631	150;150	81,062,956	12.16	5.67	0.879769
632	150;150	81,782,200	12.27	5.67	0.886708
633	150;150	80,844,620	12.13	5.64	0.880041
634	150;150	80,327,336	12.05	5.63	0.887322
635	150;150	80,238,018	12.04	5.59	0.880046
636	150;150	79,392,488	11.91	5.59	0.884198
637	150;150	78,959,294	11.84	5.59	0.876947
638	150;150	79,415,684	11.91	5.58	0.879182
639	150;150	77,956,066	11.69	5.42	0.87141
640	150;150	75,389,202	11.31	5.28	0.873075
641	150;150	74,545,228	11.18	5.19	0.880825
642	150;150	71,566,180	10.73	5.06	0.877331
643	150;150	109,086,718	32.73	15.84	0.933561
644	150;150	87,113,497	26.13	12.79	0.925379
645	150;150	110,628,480	33.19	16.2	0.931322
646	150;150	104,317,896	31.30	15.33	0.93108
647	150;150	98,716,205	29.61	14.52	0.928248
648	150;150	94,993,715	28.50	13.67	0.929069
649	150;150	87,475,637	26.24	12.83	0.924988
650	150;150	101,270,602	30.38	14.81	0.93003
651	150;150	78,533,558	23.56	11.56	0.921121
652	150;150	94,901,678	28.47	13.93	0.926848
653	150;150	94,553,703	28.37	13.88	0.92696
654	150;150	105,064,463	31.52	15.27	0.927453
655	150;150	91,874,248	27.56	13.43	0.927766
656	150;150	88,534,231	26.56	12.92	0.926383
657	150;150	85,322,254	25.60	12.36	0.923888
658	150;150	105,773,372	31.73	15.51	0.929875

659	150;150	84,140,559	25.24	12.24	0.924017
660	150;150	84,955,091	25.49	12.35	0.923029
661	150;150	85,167,785	25.55	12.48	0.923895
662	150;150	105,938,526	31.78	15.15	0.924443
663	150;150	111,009,383	33.30	16.32	0.931788
664	150;150	105,369,191	31.61	15.48	0.930346
665	150;150	110,476,630	33.14	16.11	0.930846
666	150;150	105,826,998	31.75	15.53	0.930615
667	150;150	103,082,986	30.92	15.01	0.929238
668	150;150	98,550,461	29.57	14.35	0.92747
669	150;150	87,515,666	26.25	12.8	0.925383
670	150;150	75,642,516	22.69	11.08	0.919986
671	150;150	88,454,832	26.54	12.88	0.924757
672	150;150	84,577,692	25.37	12.45	0.923505
673	150;150	90,739,161	27.22	13.22	0.927017
